# Predicting and differentiating accidental and self-harm drug poisonings using health records data

**DOI:** 10.1371/journal.pmen.0000630

**Published:** 2026-06-18

**Authors:** Gregory E. Simon, Robert D. Wellman, Susan M. Shortreed, Eric Johnson, Stacy A. Sterling, Karen J. Coleman, Brian K. Ahmedani, Zimri S. Yaseen, Andrew D. Mosholder

**Affiliations:** 1 Kaiser Permanente Washington Health Research Institute, Seattle, Washington, United States of America; 2 Department of Psychiatry and Behavioral Sciences, University of Washington School of Medicine, Seattle, Washington, United States of America; 3 Department of Biostatistics, University of Washington School of Public Health, Seattle, Washington, United States of America; 4 Kaiser Permanente Northern California Division of Research, Oakland, California, United States of America; 5 Kaiser Permanente Southern California Department of Research and Evaluation, Pasadena, California, United States of America; 6 Henry Ford Health Center for Health Services Research, Detroit, Michigan, United States of America; 7 US Food and Drug Administration, Silver Spring, Maryland, United States of America; PLOS: Public Library of Science, UNITED KINGDOM OF GREAT BRITAIN AND NORTHERN IRELAND

## Abstract

Preventing drug poisonings or overdoses will involve both generic poisoning prevention strategies and strategies specific to self-harm poisonings. This research used health records to develop statistical models predicting drug poisoning in general and self-harm poisoning in particular after an outpatient mental health specialty visit or primary care visit with mental health diagnosis. Records regarding visits from 2015 to 2019 in four health systems were used to develop models predicting poisoning following a two-step process: prediction of any poisoning or overdose followed by differentiating self-harm from accidental or undetermined intent poisoning or overdose. Separate random forest models for mental health specialty and primary care mental health visits were developed in 70% random samples of visits and validated in the remaining 30%. Among 19,130,028 visits, 114,911 (0.60%) were followed by any poisoning and 52,063 (0.27%) by self-harm poisoning. Models had moderate performance predicting any poisoning (Area under the Receiver Operating Curve or AUC = 0.778 among mental health specialty visits and 0.767 among primary care mental health visits) and good performance for specifically predicting self-harm poisoning (AUCs = 0.836 and 0.858 respectively). Mental health visits with risk above the 95^th^ percentile accounted for 39.7% of self-harm poisoning (sensitivity) and actual risk of 2.57% (positive predictive value). Predictors of any poisoning included prior self-harm, accidental poisoning, and mental health service use. Specific predictors of self-harm poisoning included age and prior mental health diagnoses or treatments. In conclusion, among outpatients with mental health diagnoses, prediction models using records data have moderate accuracy predicting any overdose or poisoning and good accuracy predicting self-harm poisoning. Self-harm and accidental poisoning have both shared and distinct predictors.

## Introduction

Overdoses or poisonings account for over 100,000 deaths annually in the US, an increase of approximately 50% over the last 5 years [1] that accelerated during the COVID-19 pandemic [2]. Nonfatal poisonings account for nearly 1,000,000 emergency department (ED) visits annually [3], and ED visits for poisoning increased during the COVID-19 pandemic despite decreases in overall ED utilization. Approximately 70% of nonfatal poisonings and 90% of fatal poisonings are classified as accidental, with the remainder coded as self-harm or undetermined intent [4,5]. Self-harm poisonings and accidental poisonings affect overlapping populations and involve shared risk factors [6,7].

Interventions to prevent accidental poisoning and interventions to prevent self-harm poisoning may be either overlapping or distinct. Preventive interventions to reduce availability of toxic drugs or medications, such as dispensing smaller quantities, safer packaging, drug “take-back” programs, or promotion of safe drug storage, aim to prevent both accidental and self-harm poisonings [8–10]. In contrast, clinical interventions such as dialectical behavior therapy [11,12] or cognitive behavioral therapy [13] specifically aim to reduce risk of intentional self-harm.

Effective targeting of interventions to prevent either accidental or self-harm poisoning will require accurate identification of people at increased risk for specific types of poisoning events. While traditional clinical risk factors have limited accuracy in predicting self-harm [14], prediction models developed from health records data can accurately identify those at risk of fatal or non-fatal self-harm [15,16]. Large health systems have begun to implement those prediction models to prompt outreach or notify treating clinicians [17,18]. In parallel, prediction models using health records data can accurately identify risk of poisoning coded as accidental [19–21]. Previous research has not, however, evaluated overlap of and differences between prediction of poisonings in general and self-harm poisonings in particular.

Here we use records data from four large health systems to examine similarities and differences between prediction of medication or drug poisoning in general and self-harm poisoning in particular. We examine two specific questions:

1) How well do statistical models developed using health records data perform in sequentially predicting any medication or drug poisoning followed by distinguishing self-harm poisonings from poisonings with accidental or undetermined intent?
2) How do the predictors of any medication or drug poisoning visit differ from the predictors differentiating self-harm poisonings from those with accidental or undetermined intent?

We focus on specialty mental health specialty visits and primary care visits with mental health diagnosis, because over half of suicide deaths and half of nonfatal suicide attempts are preceded by one of those visits within three months [22,23]. We focus on the 90 days following a health care visit, since that is the period in which potential interventions (such as safer dispensing of medications or psychotherapy addressing risk of self-harm) might take effect.

## Methods

### Ethics statement

The Kaiser Permanente institutional review board approved use of deidentified records data for this research and waived the requirement for individual informed consent. Records data were accessed between 4/16/2021 and 9/26/2022. Investigators had no access to identifying information.

### Setting and population

The four participating health systems (Henry Ford Health and the Northern California, Southern California, and Washington regions of Kaiser Permanente) provide health insurance coverage and comprehensive primary care and mental health care to a combined population of approximately 11 million members enrolled through employer-sponsored insurance, individual insurance, subsidized insurance exchange programs, Medicare, and Medicaid. Membership of each health system is representative of its service area population in terms of age, sex, race, and ethnicity [24]. Each participating health system maintains a research data warehouse integrating information from electronic health records (for all services provided by the health system) and insurance claims (for all services provided at outside facilities), including medication dispensing as well as procedure codes and diagnoses from outpatient, emergency department, and inpatient encounters [25]. Membership records are linked to state mortality data to ascertain date and cause of death for all members.

The sample included specialty mental health visits and primary care visits with mental health or substance use diagnoses beginning 10/1/2015 (when the ICD-10-CM coding system was implemented) and ending 12/31/2019 (avoiding the disruptions in care during the COVID-19 pandemic) by health system members aged 11 years or older, including all eligible visits for any individual. To ensure adequate ascertainment of outcomes, visits by patients disenrolling from the health system within 90 days were excluded.

### Outcomes

Nonfatal medication or drug poisoning events over 90 days following each eligible visit were identified by ICD-10-CM encounter diagnoses in the range T36-T65 (all poisonings with substances “chiefly medicinal in nature”) recorded at any encounter (including outpatient, emergency department, or inpatient encounters) over 90 days following an eligible visit. This included poisonings or overdoses involving prescribed and non-prescribed drugs, including poisonings with opioids, cocaine, methamphetamine, and other stimulants. Poisonings with self-harm, accidental, and undetermined intent were identified by relevant modifiers, and poisonings coded as assault were excluded. Previous research in these health systems supports the accuracy of ICD-10-CM encounter diagnoses for identifying opioid poisonings [26] and self-harm poisonings [27]. Fatal poisonings were identified from state mortality records using cause of death codes for poisoning (T36-T65) and related codes for intent, including X60-X69 (self-harm), X40-X49 (accidental), and Y10-Y19 (undetermined intent). For both nonfatal and fatal poisonings, a single event with diagnoses indicating both self-harm intent and accidental/undetermined intent was considered self-harm. For each sampled visit, classification of outcomes considered the first poisoning event during the outcome period.

### Predictors

Following methods developed in our previous research [28], approximately 2500 potential predictors of poisoning events included sociodemographic and clinical characteristics were extracted from research data warehouses for up to 60 months prior to each eligible visit. Sociodemographic characteristics included age, sex, self-reported race, self-reported Hispanic ethnicity, and neighborhood income and educational attainment from linkage of residential address to census data. Race and ethnicity data were typically reported in response to standard categories during outpatient visit registration and could be missing because a relevant visit did not occur, because questions were not asked or because a patient declined to answer. Mental health diagnoses were represented in 11 categories (e.g., depressive disorder, anxiety disorder, personality disorder), substance use disorder were represented in 5 categories (e.g., alcohol use disorder, opioid use disorder), and two additional categories represented pain and traumatic brain injury diagnoses. Mental health and substance use-related utilization were represented in 5 categories (e.g., hospitalization with mental health diagnosis, emergency department visit with substance use diagnosis). Prior injury and poisoning diagnoses were represented in 12 categories (e.g., benzodiazepine poisoning coded as accidental, self-harm). For each category of diagnosis, utilization, or prior injury/poisoning, 51 separate predictors represented different potential time patterns over the prior 60 months (e.g., number of occurrences in prior 3 months, most recent month with any occurrence). Psychiatric medication dispensings were represented in 10 categories (e.g., antidepressants, second-generation antipsychotics), and opioid-related medications were represented in 16 categories. For each medication category, 29 separate predictors represented different time patterns over the prior 60 months (e.g., days’ supply in prior 12 months, most recent month with no dispensing). Responses to each item of Patient Health Questionnaire (PHQ-9) depression questionnaires [29] completed at the sampled visit were represented by 12 potential predictors. Responses to PHQ-9 questionnaires at previous visits were represented by an additional 95 potential predictors representing various combinations and time patterns of responses. Chronic medical conditions were represented by the Charlson [30,31] comorbidity score and by binary indicators for each of the 17 conditions in Charlson score, calculated for the prior 12 months.

### Training and validating two-step prediction models for poisoning outcomes

Because mental health specialty and primary care visits with mental health diagnoses represent different patient populations and different prevention opportunities, mental health specialty and primary care visit samples were analyzed separately, with each sample divided into a 70% training set and a 30% held-out validation or test set, split at the patient level [32].

Random forest models [33] predicting and differentiating poisoning events were developed using a two-step process. In the first step, a random forest model estimated among all training visits predicted probability of any drug poisoning in the following 90 days $\left({\hat{p}}_{p}\right)$. In the second step, among training visits followed by any poisoning, a random forest model predicted if the poisoning was coded as self-harm $\left({\hat{p}}_{sh|p}\right)$. The predicted probability from the second model, differentiating self-harm intent, was then calculated for all training visits. Predicted probability of self-harm poisoning among all training visits $\left({\hat{p}}_{sh}\right)$ was calculated as the product of both steps $\left({\hat{p}}_{sh}=\;{\hat{p}}_{sh|p}{\hat{p}}_{p}\right)$. The same predictors were considered at each step, and both models were estimated using random forests with probability trees [33,34]. Tuning parameters (minimum node size and number of trees) were selected for each step using five-fold cross validation in the training dataset with folds divided at the person level and parameters selected using out-of-fold AUC (details in S2 Table and S3 Table). The number of predictors considered at each decision point was set at 50, approximately the square root of the total number of predictors.

### Evaluating model performance in the validation dataset

Final random forests were applied to all the visits in the validation sets to obtain predicted probability of any poisoning $\left({\hat{p}}_{p}\right)$ and predicted probability of a poisoning coded as self-harm $\left({\hat{p}}_{sh|p}\right)$. The performance of the two-step model was evaluated by estimating the performance of ${\hat{p}}_{sh}=\;{\hat{p}}_{sh|p}{\hat{p}}_{p}$ among all validation visits, indicated by AUC as well as the sensitivity, specificity, and positive predicted value using a cut-point at the 95^th^ percentile in training data. Confidence intervals were calculated using 10,000 bootstrap iterations performed at the visit level. All analyses were conducted with R version 3.5.3 and R studio version 1.1.463 for windows. Random forests were estimated using the R package *ranger* version 0.11.2 [35].

### Variable importance for predicting and differentiating poisoning events

Variable importance based on the gini index was calculated on the training data for all predictors used in estimating the final random forest models [33]. To allow comparison between step 1 and step 2 models, variable importance measures were normalized to a maximum of 100.

## Results

### Study sample

Table 1 displays the characteristics of 13,573,632 eligible mental health specialty visits (including 91,411 followed by any poisoning and 45,340 followed by self-harm poisoning) and characteristics of 5,556,396 eligible primary care mental health visits (including 23,500 followed by any poisoning and 6,723 followed by self-harm poisoning). Characteristics of visits assigned to the training and validations datasets are shown in S1 Table.

**Table 1:** Characteristics of full visit samples, visits followed by any poisoning, and visits followed by self-harm poisoning.

	MENTAL HEALTH SPECIALTY VISITS						PRIMARY CARE VISITS, MENTAL HEALTH DX.					
	All Visits		Any Poisoning		Self-harm Poisoning		All Visits		Any Poisoning		Self-harm Poisoning	
	N	%	N	%	N	%	N	%	N	%	N	%
Visits, n (row %)	13,573,632		91,411		45,340		5,556,396		23,500		6,723	
**Female**	8,574,389	63.2	60,379	66.1	33,997	75.0	3,447,350	62.0	14,138	62.0	4,473	62.0
**Age group (years)**												
11-17	1,789,587	13.2	18,989	20.8	13,827	30.5	412,146	7.4	1,860	7.9	1,050	15.6
18-29	2,617,897	19.3	23,209	25.4	11,717	25.8	789,295	14.2	5,052	21.5	1,766	26.3
30-44	3,452,892	25.4	20,260	22.2	9,046	20.0	1,114,307	20.1	4,903	20.9	1,545	23.0
45-64	4,140,951	30.5	22,540	24.7	9,035	19.9	1,626,292	29.3	6,827	29.1	1,813	27.0
65 or older	1,572,305	11.6	6,413	7.0	1,715	3.8	1,614,356	29.1	4,858	20.7	549	8.2
Race and ethnicity*												
Asian	1,091,683	8.0	6,674	7.3	3,956	8.7	410,426	7.4	1,860	7.9	503	7.5
American Indian/Native Alaskan	123,035	0.9	1,123	1.2	595	1.3	91,015	1.6	252	1.1	86	1.3
Black/African American	1,291,981	9.5	8,018	8.8	3,878	8.6	499,307	9.0	1,903	8.1	525	7.8
Native Hawaiian/Pacific Islander	92,100	0.7	598	0.7	319	0.7	26,292	0.5	127	0.5	53	0.8
White, non-Hispanic	7,409,666	54.6	52,545	57.5	24,705	54.5	2,914,702	52.5	13,808	58.8	3,749	55.8
Hispanic ethnicity	3,618,817	26.7	24,054	26.3	12,719	28.1	1,502,807	27.0	5,229	22.3	1,725	25.7
Not recorded	329,096	2.4	1,583	1.7	868	1.9	111,847	2.0	321	1.4	82	1.2
**Insurance Type**												
Commercial group	10,773,463	79.4	72,185	79.0	36,667	80.9	3,850,345	69.3	15,916	67.7	4,979	74.1
High-deductible Health Plan	1,005,777	7.4	5,897	6.5	2,977	6.6	416,756	7.5	1,510	6.4	453	6.7
Individual coverage	2,851,482	21.0	20,576	22.5	9,370	20.7	1,643,135	29.6	7,038	29.9	1,714	25.5
Medicaid	1,604,180	11.8	13,102	14.3	6,731	14.8	617,194	11.1	3,477	14.8	1,125	16.7
Medicare	2,262,050	16.7	14,857	16.3	6,028	13.3	1,815,482	32.7	7,169	30.5	1,272	18.9
**PHQ-9 item 9 recorded at index visit**												
Not Recorded	12,673,501	93.4	85,392	93.4	42,890	94.6	5,323,430	95.8	21,857	93.0	6,165	91.7
Response: 0	690,940	5.1	3,402	3.7	937	2.1	185,846	3.3	1,111	4.7	292	4.3
Response: 1	137,887	1.0	1,351	1.5	670	1.5	31,475	0.6	300	1.3	128	1.9
Response: 2	42,380	0.3	570	0.6	388	0.9	9,700	0.2	123	0.5	68	1.0
Response: 3	28,924	0.2	696	0.8	455	1.0	5,945	0.1	109	0.5	70	1.0
**Sum of PHQ-9 Items 1–8 recorded at index visit**												
Not Recorded	12,712,159	93.7	85,624	93.7	42,952	94.7	5,344,576	96.2	22,013	93.7	6,196	92.2
Response: 0–4	203,715	1.5	789	0.9	208	0.5	40,991	0.7	154	0.7	48	0.7
Response: 5–10	301,474	2.2	1,606	1.8	532	1.2	70,011	1.3	381	1.6	116	1.7
Response: 11–15	183,380	1.4	1,278	1.4	576	1.3	49,427	0.9	378	1.6	132	2.0
Response: 16–20	117,658	0.9	1,231	1.3	593	1.3	34,735	0.6	307	1.3	124	1.8
Response: 21 or higher	55,246	0.4	883	1.0	479	1.1	16,656	0.3	267	1.1	107	1.6
**Diagnoses Recorded in Prior 5 Years**												
Anxiety	10,680,684	78.7	78,764	86.2	39,780	87.7	3,352,915	60.3	18,003	76.6	5,475	81.4
Bipolar disorder	1,728,008	12.7	25,180	27.5	13,903	30.7	318,064	5.7	4,581	19.5	1,755	26.1
Depression	9,685,684	71.4	76,279	83.4	40,735	89.8	2,775,228	49.9	16,353	69.6	5,442	80.9
Personality disorder	1,825,988	13.5	22,738	24.9	13,039	28.8	376,675	6.8	3,866	16.5	1,582	23.5
Schizophrenia spectrum disorder	610,090	4.5	8,866	9.7	4,626	10.2	112,180	2.0	1,620	6.9	607	9.0
Traumatic brain injury	510,560	3.8	6,389	7.0	2,876	6.3	224,518	4.0	1,925	8.2	531	7.9
Opioid use disorder	593,027	4.4	11,800	12.9	3,484	7.7	218,374	3.9	4,503	19.2	1,036	15.4
**Mental Health Service Use in Prior 5 Years**												
Mental health inpatient stay	3,170,389	23.4	49,327	54.0	27,138	59.9	1,118,139	20.1	11,300	48.1	3,625	53.9
Mental health emerg dept visit	5,217,477	38.4	61,589	67.4	31,741	70.0	1,947,798	35.1	15,229	64.8	4,660	69.3
Mental health outpatient visit	12,542,903	92.4	87,927	96.2	43,851	96.7	2,734,237	49.2	17,269	73.5	5,642	83.9
**Medications Dispensed in Prior 5 Years**												
Antidepressant	9,041,564	66.6	74,503	81.5	38,211	84.3	3,167,548	57.0	17,212	73.2	5,249	78.1
Benzodiazepine	5,462,908	40.2	48,051	52.6	21,837	48.2	2,104,337	37.9	13,248	56.4	3,716	55.3
1^st^ Generation antipsychotic	528,335	3.9	7,678	8.4	3,944	8.7	182,066	3.3	1,975	8.4	571	8.5
Lithium	559,311	4.1	10,223	11.2	5,448	12.0	78,934	1.4	1,481	6.3	585	8.7
2^nd^ Generation antipsychotic	2,957,906	21.8	40,117	43.9	21,760	48.0	629,711	11.3	7,614	32.4	2,861	42.6

* Individuals who reported identifying with more than one listed race and ethnicity contribute to all selected racial and ethnic subgroups.

### Validating two-step prediction models for poisoning outcomes

Table 2 summarizes classification performance of models for prediction of any drug poisoning (Step 1) or specific prediction of self-harm poisoning (Step 1 and Step 2 combined). For prediction of any poisoning, overall performance was moderate, as indicated by an AUCs of 0.778 (95% CI 0.775 – 0.781) in the mental health specialty sample and 0.767 (95% CI 0.761 – 0.773) in the primary care mental health sample. Selecting the highest-ranked 5 percent of visits would identify 31.1% (95% CI 30.5 - 31.7) of visits in the mental health specialty sample and 33.3% (95% CI 32.2 - 34.4) of visits in the primary care sample followed by any poisoning event, representing six-fold concentrations of risk. Area under the Precision-Recall Curve (AUPRC) value were approximately eight times the event rate (the expected AUPRC value if classification was no better than chance). For specific prediction of self-harm poisoning, overall performance was good, as indicated by AUCs of 0.836 (95% CI 0.833 – 0.840) in the mental health specialty sample and 0.858 (95% CI 0.850 – 0.867) in the primary care mental health sample. Selecting the highest-ranked 5% of visits would identify 39.7% (95% CI 19.7 – 40.6) of visits in the mental health specialty sample and 47.5% (95% CI 45.3 – 49.8) of visits in the primary care mental health sample followed by a self-harm poisoning – representing eight-fold and ten-fold concentrations of risk. AUPRC values were ten to twenty times the event rate (the expected AUPRC value if classification were no better than chance).

**Table 2:** Classification performance of two-step models for prediction of any poisoning event and self-harm poisoning event. Performance was assessed in validation dataset; 95% confidence intervals were constructed using 10,000 bootstrap samples.

	Event Rate	AUC (95% CI)	Brier score (95% CI)	F-score of 95^th^ %ile (95% CI)	Sensitivity of 95^th^ %ile (95% CI)	PPV of 95^th^ %ile (95% CI)	Specificity of 95th %ile (95% CI)	AUPRC (95% CI)
STEP 1 MODEL PREDICTING ANY POISONING								
Mental Health Specialty Visits	0.0067	0.778 (0.775, 0.781)	0.00623 (0.00615, 0.0063)	0.073 (0.0712, 0.0742)	0.311 (0.305, 0.317)	0.0412 (0.0403, 0.042)	0.9517 (0.9516, 0.9518)	0.044 (0.043, 0.045)
Primary care Visits	0.0042	0.767 (0.761, 0.773)	0.0040 (0.0039, 0.0040)	0.052 (0.05, 0.055)	0.333 (0.322, 0.344)	0.028 (0.027, 0.03)	0.9511 (0.9510, 0.9512)	0.034 (0.032, 0.035)
COMBINED STEP 1 AND STEP 2 MODEL PREDICTING SELF-HARM POISONING								
Mental Health Specialty Visits	0.0033	0.836 (0.833, 0.840)	0.003 (0.0031, 0.0032)	0.048 (0.047, 0.050)	0.397 (0.389, 0.406)	0.0257 (0.0250, 0.0264)	0.9511 (0.9510, 0.9514)	0.029 (0.027, 0030)
Primary care Visits	0.0012	0.858 (0.85, 0.867)	0.0011 (0.0011, 0.0012)	0.022 (0.021, 0.023)	0.475 (0.453, 0.498)	0.011 (0.011, 0.012)	0.9505 (0.9503, 0.9507)	0.024 (0.022, 0.026)

Table 3 displays calibration performance for prediction of poisoning events in the validation sample. For prediction of any poisoning (lower left), predicted risk closely approximated observed risk, and observed risk among visits above the 99.5^th^ percentile was approximately 50 times higher than among visits below the 50^th^ percentile. For prediction of self-harm poisoning, (upper right), predicted risk closely approximated observed risk across all strata, and observed risk among visits above the 99.5^th^ percentile was approximately 100 times higher than among visits below the 50^th^ percentile. Results were similar when the primary care visit model was applied to prediction of either any poisoning or self-harm poisoning in the primary care mental health visit validation sample (S4 Table).

**Table 3:** Calibration tables for performance of two-step model predicting any poisoning event and predicting self-harm poisoning in validation sample of mental health specialty visits.

	Predicting any poisoning		Predicting self-harm poisoning	
Percentile	Average predicted probability	Proportion of visits followed by an event	Average predicted probability	Proportion of visits followed by an event
99.5<	0.090	0.114	0.060	0.066
99.0-99.5	0.055	0.049	0.037	0.031
95.0-99.0	0.033	0.030	0.020	0.020
90.0-95.0	0.018	0.017	0.009	0.009
75.0-90.0	0.010	0.010	0.004	0.005
50.0-75.0	0.005	0.005	0.002	0.002
<50.0	0.002	0.002	0.001	0.001

### Variable importance for predicting and differentiating poisoning events

**Fig 1:**
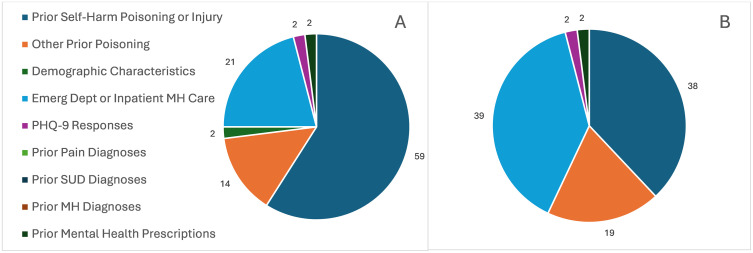
Classification of 100 most influential predictors for Step 1 model predicting any poisoning following mental health specialty visit (left) or primary care visit with mental health or substance used diagnosis (right).

**Fig 2:**
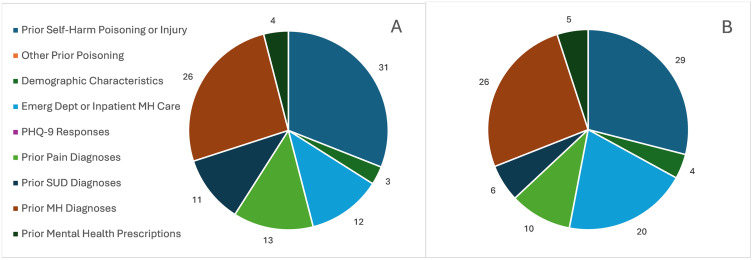
Classification of 100 most influential predictors for Step 2 model differentiating self-harm poisonings from other poisonings among mental health specialty visits (left) and general mental health visits with mental health or substance use diagnoses (right).

The left portion of Fig 1 describes the most influential predictors in the Step 1 model predicting any poisoning or overdose following a mental health specialty visit. Among the 100 most influential predictors, 59 were indicators of prior self-harm injury or poisoning, 21 were indicators of prior poisonings or overdoses coded as accidental or undetermined intent, and 14 were indicators of prior emergency department or inpatient care with mental health or substance use diagnoses. S1 Fig lists those 100 individual predictors in order of influence. The right portion of Fig 2 describes the most influential predictors in the Step 1 model predicting any poisoning or overdose following a primary care visit with mental health or substance use diagnosis. The most influential predictors were generally similar to those for mental health specialty visits, with a greater representation of indicators for emergency department or inpatient care with mental health or substance use diagnosis. S2 Fig lists those 100 individual predictors in order of influence.

The left portion of Fig 2 describes the most influential predictors in the Step 2 model differentiating self-harm overdose or poisoning from other overdose or poisoning events. The 100 most influential predictors included prior self-harm injury or poisoning, prior mental health diagnoses prior emergency department or inpatient care with mental health or substance use diagnosis, prior substance use diagnose, and prior pain diagnoses. S3 Fig lists those 100 predictors in order of influence. The right portion of Fig 2 describes parallel results for differentiating self-harm overdose or poisoning from other overdose or poisoning following a primary care visit with mental health or substance use diagnosis. Results were generally similar to those for mental health specialty visits. S4 Fig lists those 100 predictors in order of influence.

## Discussion

Prediction models developed from health records data had moderate performance in identifying patients who subsequently experienced any medication overdose or poisoning. Selecting visits in the highest 5% of the risk score distribution would identify approximately one third of visits followed by a poisoning event within 90 days – a six-fold concentration of risk. Among visits in that top 5%, the proportion actually experiencing a poisoning event in the following 90 days was approximately 1 out of 25 for mental health specialty visits and 1 out of 35 for primary care visits with mental health diagnoses.

Models using records data had greater overall classification accuracy in predicting self-harm overdose of poisoning. Selecting visits in the highest 5% of the risk score distribution would identify over 40% of visits followed by a self-harm poisoning within 90 days – an eight- to nine-fold concentration of risk. Among visits in that top 5%, the proportion actually experiencing a poisoning even in the following 90 days was approximately 1 out of 40 for mental health specialty visits and 1 out of 100 for primary care visits with mental health diagnoses. Those lower event rates or positive predictive values for self-harm poisoning reflect the lower overall prevalence of self-harm poisonings – accounting for approximately one half of all poisonings following mental health specialty visits and fewer than one-third of all poisonings following primary care visits with mental health diagnoses.

Specific predictors differentiating self-harm poisoning were moderately different from overall predictors of any overdose or poisoning. Prior self-harm events and prior use of inpatient or emergency department care for mental health or substance use diagnoses were influential as general predictors of any poisoning and as specific predictors of self-harm poisoning. Prior mental health diagnoses, prior substance use diagnoses, and prior pain diagnoses were identified as more specific predictors of self-harm poisoning or overdose. It is notable that prior self-harm injuries or poisonings were influential predictors of any poisoning event, including those coded as accidental.

Similar to previous research regarding prediction of any self-harm or suicide attempt [36], these analyses do not identify new or unexpected predictors of overdose in general or self-harm overdose. The advantage of machine learning methods lies not in discovery of new risk factors but in optimally combining and weighting known risk factors to yield more accurate composite risk scores. Neither the individual risk factors identified in Table 1 nor a simple checklist could identify risk as accurately as a high-dimensional risk score [37,38].

The predictive relationships identified by these statistical models cannot necessarily be considered causal factors or targets for intervention. Predictors should instead be considered correlates of or markers for risk. For example, we should not conclude that use of mental health medications causes increased risk of poisoning or self-harm or that use of those medications should be avoided. Instead, we would conclude that mental health medications are more often prescribed to patients already at increased risk. Similarly, we should not assume that inpatient or emergency department mental health care cause increased risk or that those services should be avoided.

Table 2 illustrates a practical trade-off between accuracy of prediction and potential public health impact. Compared to the step 1 model predicting any poisoning, the combined model predicting self-harm poisoning had superior classification accuracy as measured by AUC and sensitivity. But that more specific model had lower PPV, reflecting the lower prevalence of self-harm poisoning. Any intervention focused on patients above the 95^th^ percentile of risk in the two-step model would reach only half as many patients as an intervention guided by the broader one-step model.

Acceptable accuracy for any prediction models depends on the action or intervention that the model might inform. A positive predictive value in the range of 4%, as seen in this sample for prediction of any poisoning after a mental health specialty visit, might be appropriate for a low-intensity and low-risk intervention, such as safer packaging of medications often involved in overdose. An expected overdose or poisoning risk of 4% would likely be high enough to warrant additional clinical attention and risk assessment. Given the relatively low positive predictive value, any implementation must consider the added burden on clinical staff. But, absent a more detailed clinical assessment, that predicted rate of overdose or poisoning alone would not justify an intervention with significant risk or burden, such as involuntary hospitalization. Any clinical implementation of risk prediction scores must consider that false-positive identification of self-harm risk can have negative consequences such as unnecessary stigmatization or diverting attention from other clinical needs.

### Limitations

Interpretation of these findings should consider several limitations. Ascertainment of poisoning events was limited to poisonings that resulted in death or presented to health care settings and were formally diagnosed. Some poisonings of undetermined intent may have represented self-harm. These findings might not generalize to people without recorded mental health diagnoses or to other health systems with less complete records systems or patterns of diagnosis or service use. The discrete or coded data used in these analyses (diagnosis codes, procedure codes, medication dispensings) would not capture use of non-prescribed substances, negative life events, or social determinants of health that may significantly influence likelihood of poisoning in general or self-harm poisoning in particular [39]. Excluding visits after 12/31/2019 avoids disruptions in care during the early COVID-19 pandemic but also avoids changes in overdose patterns with increasing use of fentanyl. Implementation of this or a similar model in current practice would require testing accuracy using more recent data, possibly leading to re-calibration to account for changes in overdose rates or new model fitting to account for changes in predictive relationships.

## Conclusions

For health systems hoping to use health records data to target poisoning prevention interventions, statistical models developed using health records data have moderate accuracy in identifying patients at increased risk for any poisoning. Models developed to specifically predict self-harm poisoning have good performance, with the most influential predictors of self-harm poisoning including both prior poisoning events and indicators of prior mental health diagnosis or treatment.

## Supporting information

S1 Table Training and validation samples for mental health specialty visits and general medical visits with mental health diagnoses. (DOCX)

S2 Table Cross validation results for selecting optimal tuning parameter for random forest model predicting any poisoning among all training visits. Optimal out-of-sample AUCs are shown in bold.
(DOCX)

S3 Table Cross validation results for selecting optimal tuning parameters for random forest model predicting a self-harm diagnosed poisoning among visits followed a poisoning. Optimal out-of-sample AUCs are shown in bold parameters in bold.
(DOCX)

S4 Table Calibration tables for performance of two-step model for prediction of self-harm poisoning events in validation sample of primary care visits with mental health diagnoses. (DOCX)

S1 Fig Normalized variable importance for 100 most influential predictors in Step 1 model predicting any poisoning event in full sample of mental health specialty visits. (TIF)

S2 Fig Normalized variable importance for 100 most influential predictors in Step 1 model predicting any poisoning event in full sample of general medical visits with mental health diagnoses. (TIF)

S3 Fig Normalized variable importance for 100 most influential predictors in Step 2 model predicting self-harm poisoning, limited to mental health specialty visits followed by a poisoning event. (TIF)

S4 Fig Normalized variable importance for 100 most influential predictors in Step 2 model predicting self-harm poisoning, limited to general medical visits followed by a poisoning event. (TIF)
