# Supplementary material for: Predicting and differentiating accidental and self-harm drug poisonings using health records data
Source: PLOS Ment Health. 2026 Jun 18;3(6):e0000630. doi: 10.1371/journal.pmen.0000630 (PMC13278418; doi:10.1371/journal.pmen.0000630)
Supplement: S1 Table — (DOCX) [file pmen.0000630.s001.docx]

S1 Table - Training and validation samples for mental health specialty visits and general medical visits with mental health diagnoses

|  | Mental Health Specialty Visits | | | | General Medical Visits | | | |
| --- | --- | --- | --- | --- | --- | --- | --- | --- |
|  | Training | | Validation | | Training | | Validation | |
|  | N | % |  | % | N | %* | N | %* |
| Visits | 9,548,740 |  | 4,024,892 |  | 3,898,922 |  | 1,657,474 |  |
| Visits with any poisoning within 90 days | 65,692 | 0.6 | 25,719 | 0.6 | 9,848 |  | 4,021 |  |
| Visits with self-harm poisoning within 90 days | 32535 | 0.3 | 12805 | 0.3 | 16,823 |  | 6,677 |  |
| Unique Patients | 1,014,071 |  | 510,528 |  | 4767 |  | 1956 |  |
| Female | 6,052,195 | 63.4 | 2,522,194 | 62.7 | 2,434,340 | 62.44 | 1,013,010 | 61.12 |
| Age group (year) |  |  |  |  |  |  |  |  |
| 11-17 | 1,223,839 | 12.8 | 565,748 | 14.1 | 273,434 | 7.01 | 138,712 | 8.37 |
| 18-29 | 1,817,089 | 19.0 | 800,808 | 19.9 | 529,098 | 13.57 | 260,197 | 15.70 |
| 30-44 | 2,403,372 | 25.2 | 1,049,520 | 26.1 | 761,485 | 19.53 | 352,822 | 21.29 |
| 45-64 | 2,968,797 | 31.1 | 1,172,154 | 29.1 | 1,157,368 | 29.68 | 468,924 | 28.29 |
| 65 or older | 1,135,643 | 11.9 | 436,662 | 10.8 | 1,177,537 | 30.20 | 436,819 | 26.35 |
| Race and ethnicity* |  |  |  |  |  |  |  |  |
| Asian | 759,380 | 8.0 | 332,303 | 8.3 | 331,866 | 8.51 | 146,729 | 8.85 |
| American Indian/Native Alaskan | 89,158 | 0.9 | 33,877 | 0.8 | 36,134 | 0.93 | 15,638 | 0.94 |
| Black/African American | 910,006 | 9.5 | 381,975 | 9.5 | 348,314 | 8.93 | 145,530 | 8.78 |
| Native Hawaiian/Pacific Islander | 63,667 | 0.7 | 28,433 | 0.7 | 25,259 | 0.65 | 11,491 | 0.69 |
| White, non-Hispanic | 5,256,666 | 55.1 | 2,153,000 | 53.5 | 2,106,314 | 54.02 | 872,891 | 52.66 |
| Hispanic ethnicity | 2,522,814 | 26.4 | 1,096,003 | 27.2 | 1,072,082 | 27.50 | 462,725 | 27.92 |
| Not recorded | 219,275 | 2.3 | 109,821 | 2.7 | 74,813 | 1.92 | 41,034 | 2.48 |
| Insurance Type |  |  |  |  |  |  |  |  |
| Commercial group | 7,523,525 | 78.8 | 3,249,938 | 80.7 | 2,660,809 | 68.24 | 1,189,536 | 71.77 |
| High-deductible Health Plan | 674,144 | 7.1 | 331,633 | 8.2 | 272,096 | 6.98 | 144,660 | 8.73 |
| Individual coverage | 2,031,884 | 21.3 | 819,598 | 20.4 | 1,171,600 | 30.05 | 471,535 | 28.45 |
| Medicaid | 1,148,790 | 12.0 | 455,390 | 11.3 | 439,208 | 11.26 | 177,986 | 10.74 |
| Medicare | 1,642,945 | 17.2 | 619,105 | 15.4 | 1,326,521 | 34.02 | 488,961 | 29.50 |
| PHQ item 9 recorded at index visit |  |  |  |  |  |  |  |  |
| Not Recorded | 8,924,700 | 93.5 | 3,748,801 | 93.1 | 3,744,618 | 96.04 | 1,578,812 | 95.25 |
| Response: 0 | 478,802 | 5.0 | 212,138 | 5.3 | 123,150 | 3.16 | 62,696 | 3.78 |
| Response: 1 | 95,037 | 1.0 | 42,850 | 1.1 | 20,760 | 0.53 | 10,715 | 0.65 |
| Response: 2 | 29,804 | 0.3 | 12,576 | 0.3 | 6,475 | 0.17 | 3,225 | 0.19 |
| Response: 3 | 20,397 | 0.2 | 8,527 | 0.2 | 3,919 | 0.10 | 2,026 | 0.12 |
| Score for PHQ items 1 to 8 at index visit |  |  |  |  |  |  |  |  |
| Not Recorded | 8,948,954 | 93.7 | 3,763,205 | 93.5 | 3,757,874 | 96.38 | 1,586,702 | 95.73 |
| Response: 0-4 | 142,720 | 1.5 | 60,995 | 1.5 | 27,664 | 0.71 | 13,327 | 0.80 |
| Response: 5-10 | 208,874 | 2.2 | 92,600 | 2.3 | 46,375 | 1.19 | 23,636 | 1.43 |
| Response: 11-15 | 127,614 | 1.3 | 55,766 | 1.4 | 32,936 | 0.84 | 16,491 | 0.99 |
| Response: 16-20 | 81,804 | 0.9 | 35,854 | 0.9 | 23,123 | 0.59 | 11,612 | 0.70 |
| Response: 21 or higher | 38,774 | 0.4 | 16,472 | 0.4 | 10,950 | 0.28 | 5,706 | 0.34 |
| Diagnoses Recorded in Prior 5 Years |  |  |  |  |  |  |  |  |
| Anxiety | 7,573,746 | 79.3 | 3,106,938 | 77.2 | 2,417,519 | 62.00 | 935,396 | 56.44 |
| Bipolar | 1,269,375 | 13.3 | 458,633 | 11.4 | 232,076 | 5.95 | 85,988 | 5.19 |
| Depression | 6,900,894 | 72.3 | 2,784,790 | 69.2 | 2,026,512 | 51.98 | 748,716 | 45.17 |
| Personality disorder | 1,338,790 | 14.0 | 487,198 | 12.1 | 280,788 | 7.20 | 95,887 | 5.79 |
| Schizophrenia spectrum disorder | 449,434 | 4.7 | 160,656 | 4.0 | 81,918 | 2.10 | 30,262 | 1.83 |
| Traumatic brain injury | 366,504 | 3.8 | 144,056 | 3.6 | 160,183 | 4.11 | 64,335 | 3.88 |
| Opioid use disorder (excluding remission) | 427,916 | 4.5 | 165,111 | 4.1 | 159,119 | 4.08 | 59,255 | 3.58 |
| Opioid use disorder in remission | 211,634 | 2.2 | 82,439 | 2.0 | 39,918 | 1.02 | 14,566 | 0.88 |
| Mental Health Utilization in Prior 5 Years | | | | | | | | |
| Mental health inpatient stay | 2,303,913 | 24.1 | 866,476 | 21.5 | 822,384 | 21.09 | 295,755 | 17.84 |
| Mental health emergency department visit | 3,773,122 | 39.5 | 1,444,355 | 35.9 | 1,424,982 | 36.55 | 522,816 | 31.54 |
| Mental health outpatient visit | 8,899,679 | 93.2 | 3,643,224 | 90.5 | 1,962,052 | 50.32 | 772,185 | 46.59 |
| Medications Dispensed in Prior 5 Years | | | | | | | | |
| Antidepressant | 6,490,255 | 68.0 | 2,551,309 | 63.4 | 2,305,348 | 59.13 | 862,200 | 52.02 |
| Benzodiazepine | 3,970,972 | 41.6 | 1,491,936 | 37.1 | 1,549,553 | 39.74 | 554,784 | 33.47 |
| First generation antipsychotic | 388,769 | 4.1 | 139,566 | 3.5 | 134,797 | 3.46 | 47,269 | 2.85 |
| Lithium | 407,753 | 4.3 | 151,558 | 3.8 | 57,706 | 1.48 | 21,228 | 1.28 |
| Second generation antipsychotic | 2,173,169 | 22.8 | 784,737 | 19.5 | 461,907 | 11.85 | 167,804 | 10.12 |
| * Individuals who reported identifying with more than one listed race and ethnicity contribute to all selected racial and ethnic subgroups. | | | | | | | | |
