# Supplementary material for: Predicting and differentiating accidental and self-harm drug poisonings using health records data
Source: PLOS Ment Health. 2026 Jun 18;3(6):e0000630. doi: 10.1371/journal.pmen.0000630 (PMC13278418; doi:10.1371/journal.pmen.0000630)
Supplement: S2 Table — Optimal out-of-sample AUCs are shown in bold. (DOCX) [file pmen.0000630.s002.docx]

S2 Table - Cross validation results for selecting optimal tuning parameter for random forest model predicting any poisoning among all training visits. Optimal out-of-sample AUCs are shown in bold

| Number of trees | Terminal node size | Mental Health Specialty Visits Out-of-sample AUC | General Medical Visits  Out-of-sample AUC |
| --- | --- | --- | --- |
| 10 | 1,000 | 0.7242 | 0.7314 |
| 10 | 10,000 | 0.7593 | 0.7616 |
| 10 | 25,000 | 0.7663 | 0.7693 |
| 10 | 50,000 | 0.7706 | 0.7690 |
| 10 | 100,000 | 0.7712 | 0.7689 |
| 10 | 200,000 | 0.7730 | 0.7661 |
| 100 | 1,000 | NA* | 0.7604 |
| 100 | 10,000 | 0.7721 | 0.7733 |
| 100 | 25,000 | 0.7768 | **0.7762** |
| 100 | 50,000 | 0.7775 | 0.7758 |
| 100 | 100,000 | **0.7781** | 0.7738 |
| 100 | 200,000 | 0.7762 | 0.7703 |
| *Not estimated because computational burden too high. | | | |
