# Supplementary material for: Predicting and differentiating accidental and self-harm drug poisonings using health records data
Source: PLOS Ment Health. 2026 Jun 18;3(6):e0000630. doi: 10.1371/journal.pmen.0000630 (PMC13278418; doi:10.1371/journal.pmen.0000630)
Supplement: S3 Table — Optimal out-of-sample AUCs are shown in bold parameters in bold. (DOCX) [file pmen.0000630.s003.docx]

S3 Table - Cross validation results for selecting optimal tuning parameters for random forest model predicting a self-harm diagnosed poisoning among visits followed a poisoning. Optimal out-of-sample AUCs are shown in bold parameters in bold

| Number of trees | Terminal node size | Mental Health Specialty Visits  Out-of-sample AUC | General Medical Visits  Out-of-sample AUC |
| --- | --- | --- | --- |
| 10 | 10 | 0.723 | 0.728 |
| 10 | 100 | 0.734 | 0.741 |
| 10 | 1,000 | 0.759 | 0.752 |
| 10 | 10,000 | 0.744 | 0.699 |
| 10 | 25,000 | 0.716 | 0.471 |
| 10 | 50,000 | 0.679 | 0.472 |
| 100 | 10 | 0.767 | 0.471 |
| 100 | 100 | 0.766 | 0.759 |
| 100 | 1,000 | **0.770** | 0.764 |
| 100 | 10,000 | 0.752 | **0.765** |
| 100 | 25,000 | 0.728 | 0.709 |
| 100 | 50,000 | 0.690 | 0.471 |
