# Supplementary material for: Predicting and differentiating accidental and self-harm drug poisonings using health records data
Source: PLOS Ment Health. 2026 Jun 18;3(6):e0000630. doi: 10.1371/journal.pmen.0000630 (PMC13278418; doi:10.1371/journal.pmen.0000630)
Supplement: S4 Table — (DOCX) [file pmen.0000630.s004.docx]

S4 Table - Calibration tables for performance of two-step model for prediction of self-harm poisoning events in validation sample of primary care visits with mental health diagnoses

|  | Predicting any poisoning | | Predicting self-harm poisoning | |
| --- | --- | --- | --- | --- |
| Percentile | Average predicted probability | Proportion of visits followed by an event | Average predicted probability | Proportion of visits followed by an event |
| 99.5< | 0.077 | 0.075 | 0.038 | 0.036 |
| 99.0-99.5 | 0.045 | 0.046 | 0.021 | 0.018 |
| 95.0-99.0 | 0.023 | 0.020 | 0.008 | 0.007 |
| 90.0-95.0 | 0.011 | 0.010 | 0.003 | 0.004 |
| 75.0-90.0 | 0.006 | 0.006 | 0.001 | 0.001 |
| 50.0-75.0 | 0.003 | 0.003 | 0.001 | 0.001 |
| <50.0 | 0.001 | 0.001 | 0.000 | 0.000 |
