# Supplementary figures and images for: Predicting and differentiating accidental and self-harm drug poisonings using health records data
Source: PLOS Ment Health. 2026 Jun 18;3(6):e0000630. doi: 10.1371/journal.pmen.0000630 (PMC13278418; doi:10.1371/journal.pmen.0000630)

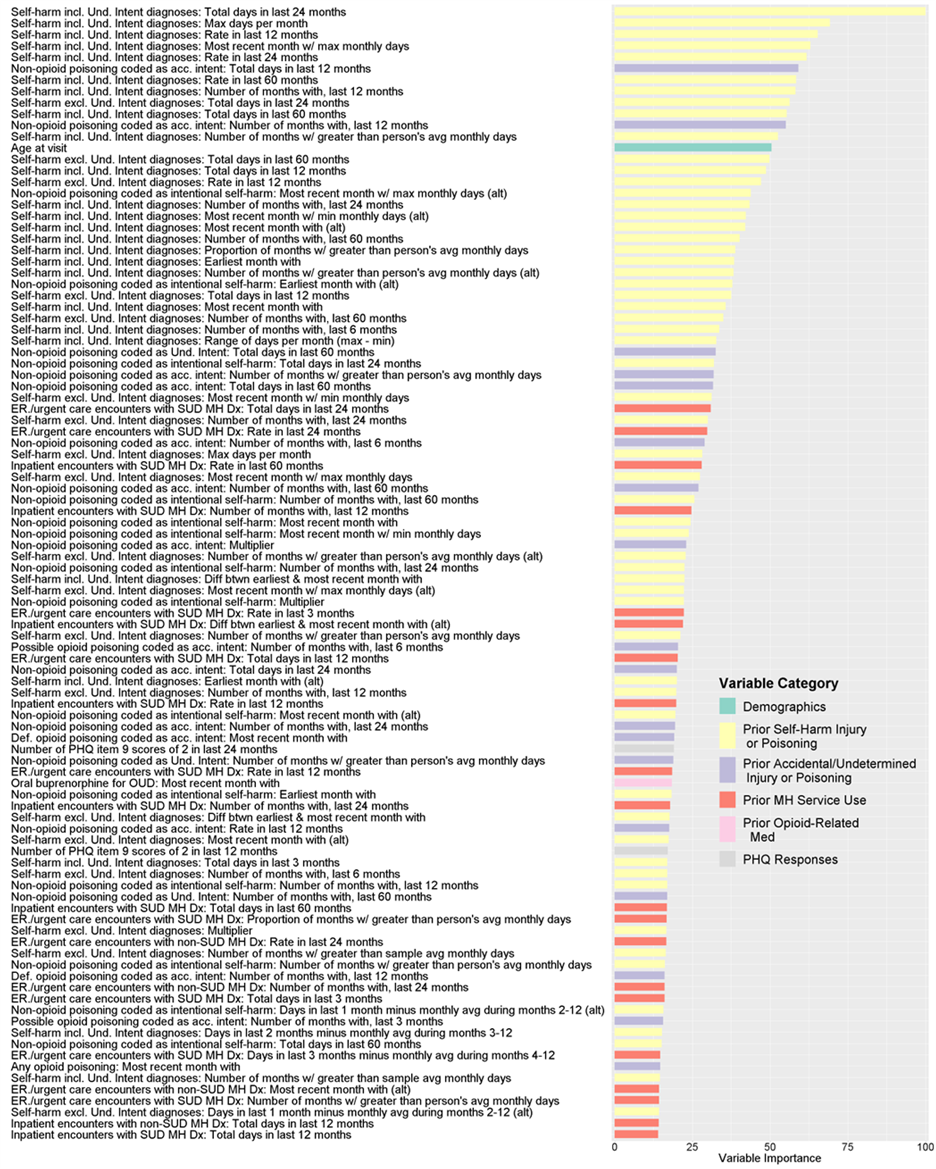

Supplement: S1 Fig — (TIF) [file pmen.0000630.s005.tif]

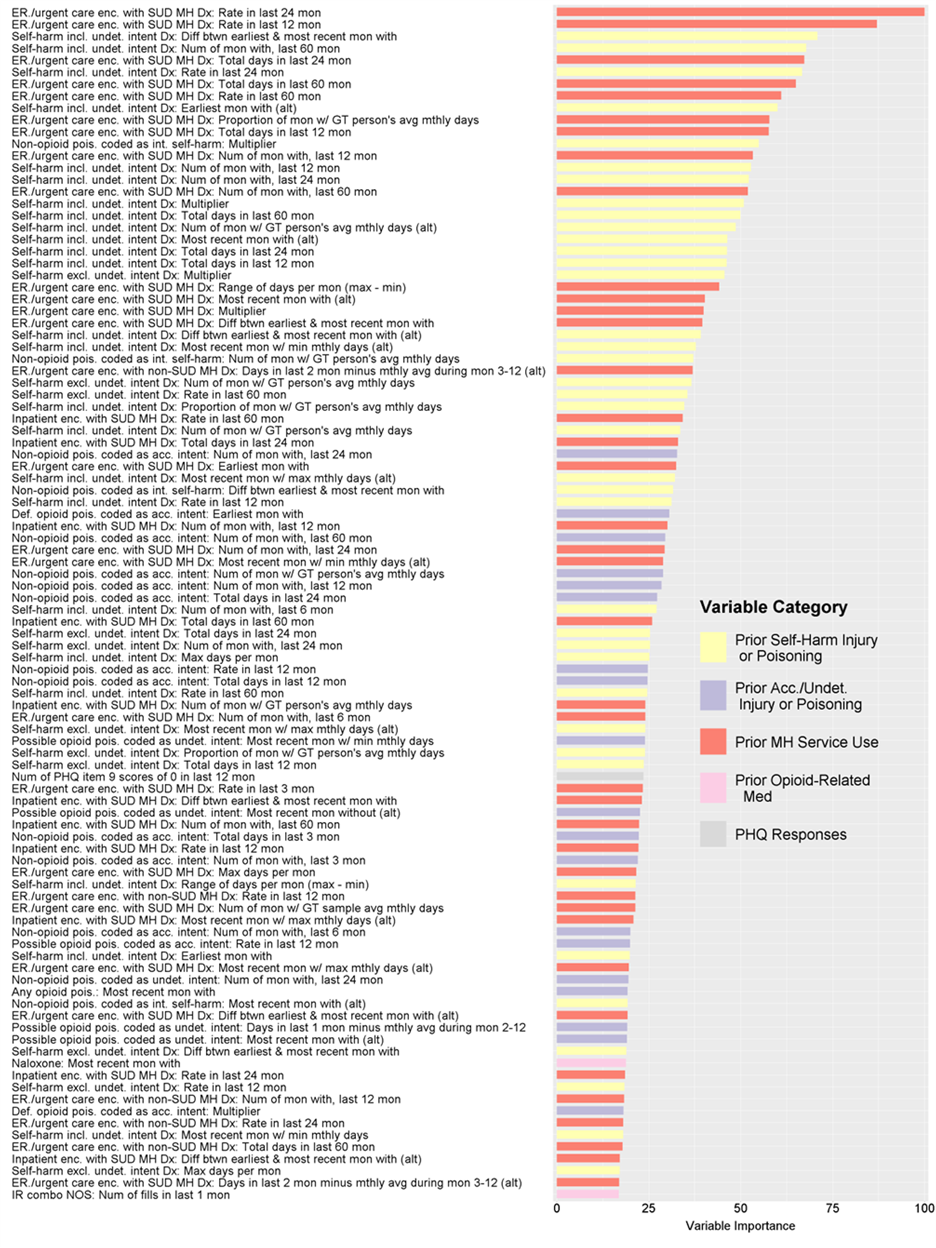

Supplement: S2 Fig — (TIF) [file pmen.0000630.s006.tif]

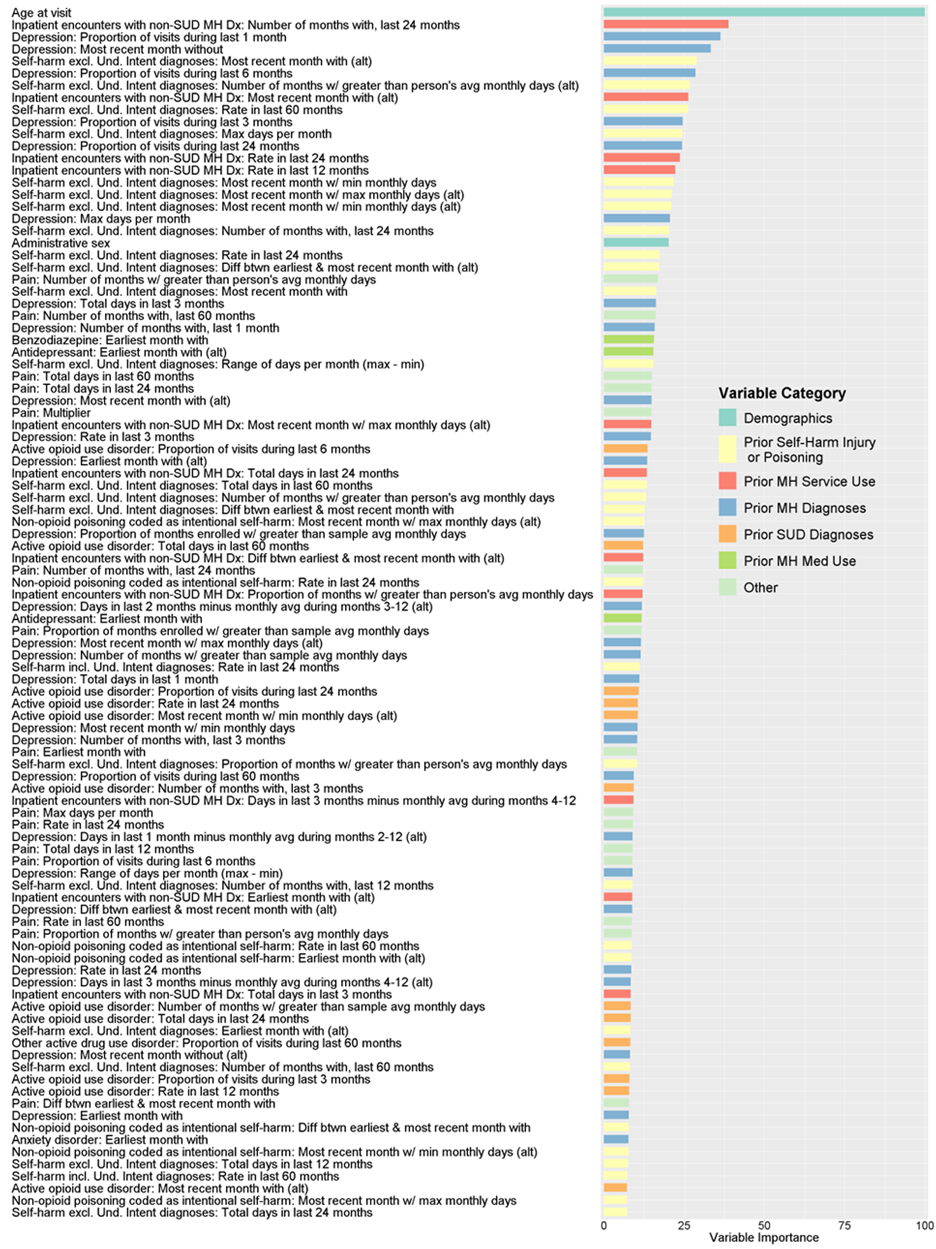

Supplement: S3 Fig — (TIF) [file pmen.0000630.s007.tif]

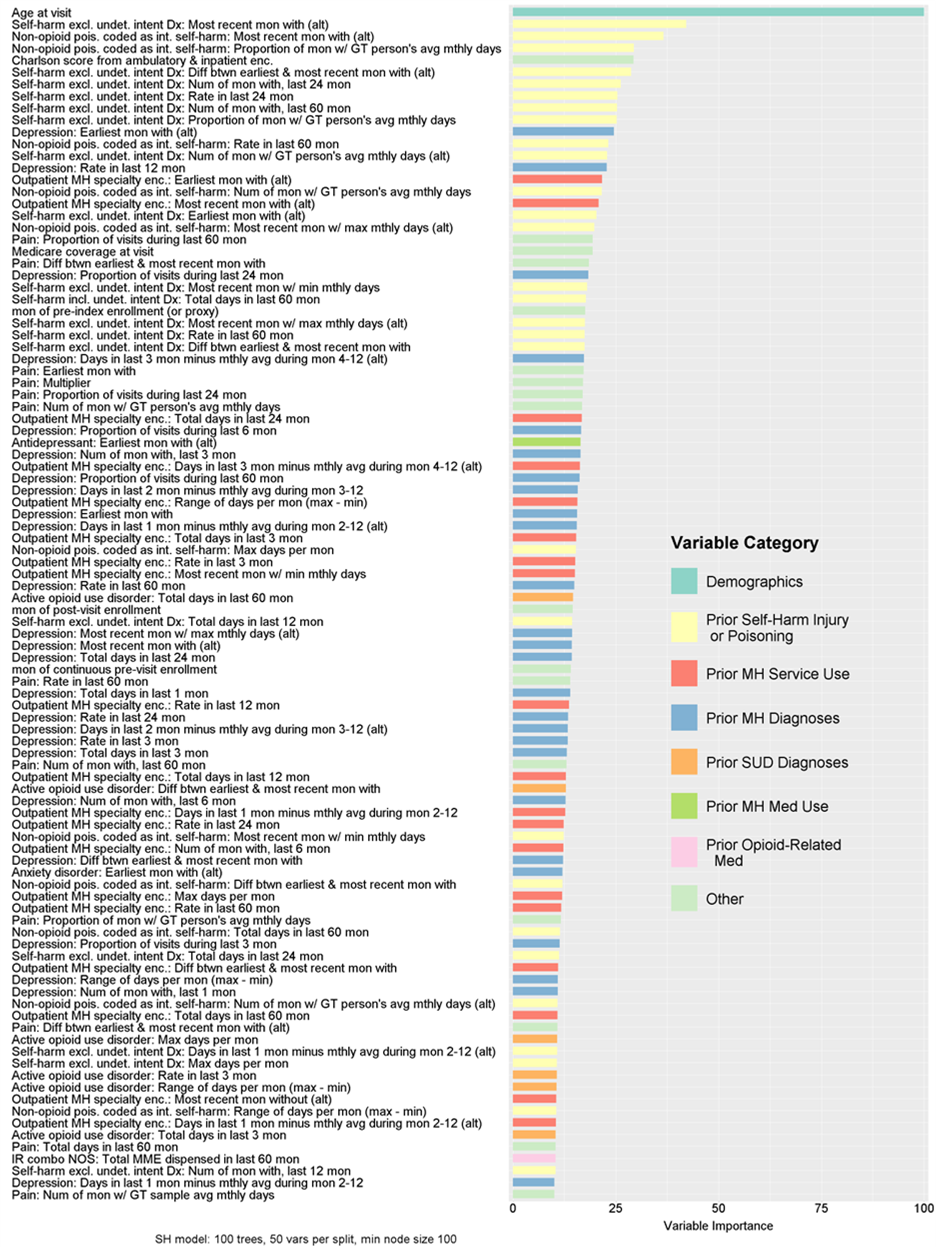

Supplement: S4 Fig — (TIF) [file pmen.0000630.s008.tif]
