# Supplementary material for: Predicting and differentiating accidental and self-harm drug poisonings using health records data
Source: PLOS Ment Health. 2026 Jun 18;3(6):e0000630. doi: 10.1371/journal.pmen.0000630 (PMC13278418; doi:10.1371/journal.pmen.0000630)
Supplement: Attachment — Submitted filename: response to PLOS MH reviews.docx [file pmen.0000630.s010.docx]

Responses to Editor comments

1) Provide exact wording for disclosure statement – This provided on page 2.
2) List all sources of financial support, including grant numbers – This is provided on page 2
3) Identify specific recipients of funding – This is provided on page 2. Simon was the principal investigator/primary awardee for the FDA contract, but several other authors were supported by this contract. Please advise if only Simon should be listed as the grant recipient
4) Describe role of funders in the study – This is provided on page 2
5) State if any authors received a salary from a funder – This is provided on page 2 (two authors were employees of the funder).
6) Include a separate Ethics Statement at the beginning of the Methods section – This information is now specifically called out at the beginning of the Methods section (page 7, line 109)
7) Provide legends for supporting information – A legend or table of contents has been added to the first page of the appendix document. Please advise if this is not what you were requesting.
8) Can data be shared on request? – Unfortunately, the individual-level data used in these analyses cannot be shared. These data concern include details regarding sensitive and highly stigmatized events (drug overdoses, suicide attempts, psychiatric hospitalizations), and health system patient did not provide informed consent for use of these data in research. While data do not contain any explicit identifiers (e.g. name, birth date, medical record number, etc.), these data consider relatively rare events that could be ascertained from other sources Combinations of individual characteristic (age group, race, ethnicity, state of residence) could be used to isolate and reidentify individual patients. This risk is discussed in detail in one of our previous publications, using very similar data as an example ([Assessing and Minimizing Re-identification Risk in Research Data Derived from Health Care Records - PubMed](https://pubmed.ncbi.nlm.nih.gov/30972355/)). Mindful of this risk, the Kaiser Permanente IRB approved research use of these data under the condition that individual data would not be shared. Subsequent events (including $50 million settlement of a large class-action lawsuit against Kaiser Permanente regarding sharing of data thought to be deidentified) have created an even stricter climate regarding any sharing of data derived from patient records. I would be happy to discuss this issue directly with the editor if that would be helpful.

Responses to Reviewer comments

1) Fix typographical errors on pages 3 and 14 – Now corrected.
2) Clarify the nature of the study sample – We have made minor changes in several locations throughout the abstract and text to clarify that the primary care sample included only visits with mental health or substance use diagnoses. We have also added text to the end of introduction (page 6, line 100) justifying this choice (the majority of suicide deaths and the majority of suicide attempts are preceded by one of these types of visits).
3) Revise Figures – Figures 1 and 2 are now presented as bar graphs, and we agree with the reviewer that this presentation is clearer. We intentionally chose to use the same categories and color scheme in Figures 1 and 2 so that readers could more easily see similarities and differences between the most influential predictors for these two tasks (general prediction of overdose, differentiation of self-harm overdose). Some categories are not present in Figure 1, but we believe it is informative to show explicitly that those categories are not represented (by including categories with zero members).
4) Report specificity and emphasize potential harms of false positive identification – Table 2 now reports specificity values for a cut-point at the 95^th^ %ile of the risk score distribution. We have also added text to the discussion (page 17, line 338) regarding potential negative consequences of false-positive identification. The text description of results and the discussion, however, do not emphasize specificity. While we agree with the reviewer regarding this issue (adverse consequences of false-positive identification), we believe that emphasizing specificity can give a misleading impression of high accuracy in the case of rare outcomes. As seen in Table 2, specificity values all exceeded 95% despite PPV values below 5%. Furthermore, specificity values were nearly identical for scenarios with PPV values ranging from 1% to 4%. In this case, a “high” specificity is more a reflection of a low outcome base rate rather than high model performance.
5) Explain/justify selection of 90 day outcome window – We have added text to the end of the introduction (page 6, para 102) explaining this choice. The 90-day window following a health care contact is the time during which low- and moderate-intensity interventions to reduce risk (such as psychotherapy focused on self-harm risk OR safer dispensing of drugs that might be used in overdose) would take effect.
6) Should models consider suicide mortality or fatal self-harm? – Both of the outcomes considered (any poisoning or overdose and self-harm poisoning or overdose) include both nonfatal events (ascertained from health system records) and fatal events (ascertained from state mortality data). Fortunately, only a small proportion of overdose events are fatal. In this sample, the number of fatal self-harm overdoses in this sample is too small to support the detailed analyses presented here. While our previous work has considered prediction of fatal self-harm, that work included fatal self-harm injuries (such as suicide by firearms, hanging, and jumping) which account for the majority of suicide deaths.
