# Supplementary material for: Predicting and differentiating accidental and self-harm drug poisonings using health records data
Source: PLOS Ment Health. 2026 Jun 18;3(6):e0000630. doi: 10.1371/journal.pmen.0000630 (PMC13278418; doi:10.1371/journal.pmen.0000630)
Supplement: Attachment — Submitted filename: response to 2nd PLOS review.docx [file pmen.0000630.s011.docx]

Dear Editors –

We thank you and the journal’s reviewers for your attention to our manuscript. We have prepared a revised version in which we attempt to address the most recent questions and suggestions. Our specific responses are listed below, with page and paragraph numbers referring to the “clean” version of the revised manuscript.

1) Please report number of overlapping cases (events with diagnoses of self-harm and accidental or undetermined intent) – We have revised the description of Methods to more clearly describe the assignment of diagnoses (page 8, para 1), but we are not able to provide the detail the reviewer requests. Our original data extraction used a hierarchical rule (self-harm intent supersedes accidental or undetermined intent) to classify each event and did not retain all diagnostic codes for every encounter. One event could generate multiple encounters and multiple diagnoses per encounter across more than one day. We did not (and do not) have access to those more detailed (and potentially identifiable) data.
2) Please expand on how updating might be necessary to adapt to changes in predictors or predictive relationships – The revised Discussion (page 18, para 1) now mentions the possible need for re-calibration or new model fitting.
3) Please expand on the implications of relatively low PPV for clinical burden – The revised discussion (page 17, para 1) specifically mentions the need to consider burden on clinical staff.
4) Please report AUPRC as an alternative indicator of model performance – We have added this information to Table 2 and refer to it in the revised Results section (pages 13-14). Because AUPRC cannot really be interpreted independent of event rate, we have added that information to Table 2.
5) Consider mentioning how random forest models account for interactions and nonlinear relationships – We agree with the reviewer that random forest models have the potential to improve prediction bye accounting for interactions and nonlinear relationships, but we do not believe that potential applies directly to this work predicting overdose or self-harm. Our previous work using these same data (PMC10036475) found that random forest models allowing complex interaction and much more detailed temporal encoding of predictors did not significantly improve prediction of self-harm or suicide attempt compared to simpler penalized logistic models.
6) State clearly that utilization predictors are not causal targets – We have added to the discussion regarding this issue, specifically mentioning inpatient or emergency department mental health care (page 16, para 2).
7) Can the most influential predictors be summarized in a table in the main manuscript? - We have prepared the requested table and included it in this revision package. But we do not believe it would be helpful to journal readers and we recommend against including it in the main manuscript. Our approach to feature engineering was to include diverse and highly detailed temporal encoding for each predictor domain (typically 50 or more temporal patterns). Given the random nature of random forest models, the most influential predictors typically included a large number of very closely related predictors. For example, the 15 most influential predictors of any poisoning after a mental health specialty visit included 11 different temporal encodings of prior self-harm diagnoses. We believe that the graphical presentation (pie charts showing different domains of predictors) will be most informative for most readers. Readers interested in detail can view the more detailed graphical presentations in the appendix (showing the 100 most influential predictors in each model).
