# Supplementary material for: Predicting and differentiating accidental and self-harm drug poisonings using health records data
Source: PLOS Ment Health. 2026 Jun 18;3(6):e0000630. doi: 10.1371/journal.pmen.0000630 (PMC13278418; doi:10.1371/journal.pmen.0000630)
Supplement: Peer review history

# Peer review: Decision Letter 0

**DOI:** 10.1371/journal.pmen.0000630.r001

**Authors:** Jason Morgan

18 Jan 2026

PMEN-D-25-00516

Predicting and differentiating accidental and self-harm drug poisonings using health records data

PLOS Mental Health

Dear Dr. Simon,

Thank you for submitting your manuscript to PLOS Mental Health. After careful consideration, we feel that it has merit but does not fully meet PLOS Mental Health’s publication criteria as it currently stands. Therefore, we invite you to submit a revised version of the manuscript that addresses the points raised during the review process.

Your manuscript has been assessed by one reviewers. While they indicate some interest in your work, they have raised several concerns that require your attention. Please consider these points carefully when preparing your revised manuscript and point-by-point response document.

Please note that we have only been able to secure a single reviewer to assess your manuscript. We are issuing a decision on your manuscript at this point to prevent further delays in the evaluation of your manuscript. Please be aware that the editor who handles your revised manuscript might find it necessary to invite additional reviewers to assess this work once the revised manuscript is submitted. However, we will aim to proceed on the basis of this single review if possible.

Please submit your revised manuscript by Feb 28 2026 11:59PM. If you will need more time than this to complete your revisions, please reply to this message or contact the journal office at mentalhealth@plos.org. When you're ready to submit your revision, log on to https://www.editorialmanager.com/pmen/ and select the 'Submissions Needing Revision' folder to locate your manuscript file.

Please include the following items when submitting your revised manuscript:

- A letter that responds to each point raised by the editor and reviewer(s). You should upload this letter as a separate file labeled 'Response to Reviewers'.
- A marked-up copy of your manuscript that highlights changes made to the original version. You should upload this as a separate file labeled 'Revised Manuscript with Track Changes'.
- An unmarked version of your revised paper without tracked changes. You should upload this as a separate file labeled 'Manuscript'.

Guidelines for resubmitting your figure files are available below the reviewer comments at the end of this letter.

We look forward to receiving your revised manuscript.

Kind regards,

Jason Morgan

Staff Editor

PLOS Mental Health

Journal Requirements:

1. Please amend your detailed Financial Disclosure statement. This is published with the article. It must therefore be completed in full sentences and contain the exact wording you wish to be published.

i. Please clarify all sources of financial support for your study. List the grants, grant numbers, and organizations that funded your study, including funding received from your institution. Please note that suppliers of material support, including research materials, should be recognized in the Acknowledgements section rather than in the Financial Disclosure.

ii. State the initials, alongside each funding source, of each author to receive each grant. For example: "This work was supported by the National Institutes of Health (####### to AM; ###### to CJ) and the National Science Foundation (###### to AM)."

iii. State what role the funders took in the study. If the funders had no role in your study, please state: “The funders had no role in study design, data collection and analysis, decision to publish, or preparation of the manuscript.”

iv. If any authors received a salary from any of your funders, please state which authors and which funders.

2. Please ensure that your Ethics Statement is available in its entirety at the beginning of your Methods section, under a subheading 'Ethics Statement'. It must include:

i) The name(s) of the Institutional Review Board(s) or Ethics Committee(s)

ii) The approval number(s), or a statement that approval was granted by the named board(s)

iii) (for human participants or donors) - A statement that formal consent was obtained (must state whether verbal/written) OR the reason consent was not obtained (e.g., anonymity).

3. We have noticed that you have uploaded Supporting Information files, but you have not included a list of legends. Please add a full list of legends for your Supporting Information files after the references list.

4. We note that you have indicated that there are restrictions to data sharing for this study. For studies involving human research participant data or other sensitive data, we encourage authors to share de-identified or anonymized data. However, when data cannot be publicly shared for ethical reasons, we allow authors to make their data sets available upon request. For information on unacceptable data access restrictions, please see http://journals.plos.org/plosone/s/data-availability#loc-unacceptable-data-access-restrictions.

Before we proceed with your manuscript, please address the following prompts:

a) If there are ethical or legal restrictions on sharing a de-identified data set, please explain them in detail (e.g., data contain potentially identifying or sensitive patient information, data are owned by a third-party organization, etc.) and who has imposed them (e.g., a Research Ethics Committee or Institutional Review Board, etc.). Please also provide contact information for a data access committee, ethics committee, or other institutional body to which data requests may be sent.

b) If there are no restrictions, please upload the minimal anonymized data set necessary to replicate your study findings to a stable, public repository and provide us with the relevant URLs, DOIs, or accession numbers. Please see http://www.bmj.com/content/340/bmj.c181.long for guidelines on how to de-identify and prepare clinical data for publication. For a list of recommended repositories, please see https://journals.plos.org/plosone/s/recommended-repositories. You also have the option of uploading the data as Supporting Information files, but we would recommend depositing data directly to a data repository if possible.

Please update your Data Availability statement in the submission form accordingly.

If the reviewer comments include a recommendation to cite specific previously published works, please review and evaluate these publications to determine whether they are relevant and should be cited. There is no requirement to cite these works unless the editor has indicated otherwise.

Additional Editor Comments (if provided):

[Note: HTML markup is below. Please do not edit.]

Reviewers' comments:

Reviewer's Responses to Questions

**Comments to the Author**

1. Does this manuscript meet PLOS Mental Health’s publication criteria? Is the manuscript technically sound, and do the data support the conclusions? The manuscript must describe methodologically and ethically rigorous research with conclusions that are appropriately drawn based on the data presented.

Reviewer #1: Yes

**********

2. Has the statistical analysis been performed appropriately and rigorously?

Reviewer #1: Yes

**********

3. Have the authors made all data underlying the findings in their manuscript fully available (please refer to the Data Availability Statement at the start of the manuscript PDF file)?

The PLOS Data policy requires authors to make all data underlying the findings described in their manuscript fully available without restriction, with rare exception. The data should be provided as part of the manuscript or its supporting information, or deposited to a public repository. For example, in addition to summary statistics, the data points behind means, medians and variance measures should be available. If there are restrictions on publicly sharing data—e.g. participant privacy or use of data from a third party—those must be specified.

Reviewer #1: No

**********

4. Is the manuscript presented in an intelligible fashion and written in standard English?

PLOS Mental Health does not copyedit accepted manuscripts, so the language in submitted articles must be clear, correct, and unambiguous. Any typographical or grammatical errors should be corrected at revision, so please note any specific errors here.

Reviewer #1: Yes

**********

5. Review Comments to the Author

Please use the space provided to explain your answers to the questions above. You may also include additional comments for the author, including concerns about dual publication, research ethics, or publication ethics. (Please upload your review as an attachment if it exceeds 20,000 characters)

Reviewer #1: Thak you for the opportunity to review this paper demonstrating the modelling of poisoning, including accidental vs intentional poisoning. The paper is generally sound, although I would like the authors to consider some issues.

First, I spotted two minor typos:

P3L38 add “to”

P14L281 change “even tin” to “event in”

There are some major issues I would like the authors to consider.

First, there is lack of clarity about the sample. P7L115–121 notes: “The sample included specialty mental health visits and primary care visits with mental health or substance use diagnoses… including all eligible visits for any individual.” So first, is an “eligible visit” a mental health visit (either specialty or primary care with mental health/substance use)? Does this mean that all other clients were excluded? From Table 1 (which is too late to be certain about this point), it seems so. If so, this should probably be reflected in the title and abstract, as the underlying populations are quite different. The abstract only mentions “after an outpatient mental health or primary care visit”, which is ambiguous. Obviously, a mental health/substance use visit is, in and of itself, a predictor of subsequent drug poisoning. So: there are two different research questions for the two different populations: “What are predictors (one of which is possibly mental health/substance use visit) of drug poisoning in a general population?” vs “What are predictors of drug poisoning in a population of patients who have had a mental health/substance use visit?” I gather that it is the latter which the authors are investigating, but this should be made clear right from the title onward. Generally, I think the authors should also more clearly define the term “eligible visit” in the context of their study. They give the ICD codes for the outcomes, but very little detail for defining the eligible visits.

Depending on what exactly is their underlying population, the authors could also, if their underlying population is only those patients with specialty mental health or primary care with mental health/substance use), rephrase sentences like (P9L173): “Because mental health specialty and primary care visits” to “Because specialty- and primary care mental health visits” in order to avoid confusion.

Second, please redo both figures. Pie charts are almost never a good graphical choice (https://theconversation.com/heres-why-you-should-almost-never-use-a-pie-chart-for-your-data-214576), and I’m afraid this paper is one more example of where they are not. In fact, if the authors chose a simple table, Figures 1 and 2 could be collapsed into a single, more readable, entity. And if they insist on using figures, then four stacked bar charts in a 2x2 small-multiple chart could also collapse them into a single entity. Regardless, the legend shows that there are nine categories, and yet the charts only show six or seven. Even in a combined bar chart as suggested, the authors should ensure that all nine category value labels are displayed (in a table, this is not a problem).

Third, I am glad that the authors note that “The predictive relationships identified by these statistical models cannot necessarily be considered causal factors or targets for intervention.” (P15L303–4). I think the discussion of this on P16 is quite good. However, I would like the authors to more explicitly consider the issue of Specificity. What are the implications, in this context, of not correctly identifying the patients who do not require an intervention. Commendably, the authors recommend at most “low-intensity and low-risk intervention” (P16L320). On P10L197 the authors mention specificity, but it is never reported nor discussed. The issue here might be illustrated with the similar situation in breast cancer screening. Better screening leads to more cases being identified, but also leads to greater numbers of people receiving treatment unnecessarily. In breast cancer screening, emphasis is put on the NNT and NNH. The point here is that interventions, even low intensity and low risk, can have unintended consequences. The authors rightly point out that their model is not enough to justify interventions alone. But one has to ask, then, what the point of the model is? This paper, in my opinion, needs comprehensive reporting showing not only how many patients are correctly identified as being at risk of future poisoning, but also how many patients at risk were missed, how many patients deemed not at risk did actually experience a subsequent poisoning event, and how many patients deemed at risk did not poisoning (i.e., reporting that covers all four quadrants of the confusion matrix). At present, the paper only considers sensitivity and PPV.

This leads to the last suggestion, which relates to the scope of the paper. First, no justification is given for the 90-day follow-up period. There is some literature to indicate that indeed, most repeat poisonings do occur quite soon after the index, but the authors here are not investigating repeat poisoning. I don't think 90 days is necessarily wrong, just that it should be justified. And it should also be recommended, I think, that more work be done on slightly longer time periods. Also, in terms of scope, it was interesting to note that the authors recorded mortality (P8L133-4), but did not examine the performance of their model considering that outcome. It is not a faultless distinction, but when examining poisoning populations, there is a clear distinction and clear population demographic differences between those with suicidal intent and those who are self-harming.

**********

6. PLOS authors have the option to publish the peer review history of their article (what does this mean?). If published, this will include your full peer review and any attached files.

**Do you want your identity to be public for this peer review?**  If you choose “no”, your identity will remain anonymous but your review may still be made public.

For information about this choice, including consent withdrawal, please see our Privacy Policy.

Reviewer #1: **Yes:**  Jacques Eugene Raubenheimer

**********

[NOTE: If reviewer comments were submitted as an attachment file, they will be attached to this email and accessible via the submission site. Please log into your account, locate the manuscript record, and check for the action link "View Attachments". If this link does not appear, there are no attachment files.]

-->Figure Resubmissions:

While revising your submission, we strongly recommend that you use PLOS’s NAAS tool (https://ngplosjournals.pagemajik.ai/artanalysis) to test your figure files. NAAS can convert your figure files to the TIFF file type and meet basic requirements (such as print size, resolution), or provide you with a report on issues that do not meet our requirements and that NAAS cannot fix.

After uploading your figures to PLOS’s NAAS tool - https://ngplosjournals.pagemajik.ai/artanalysis, NAAS will process the files provided and display the results in the "Uploaded Files" section of the page as the processing is complete. If the uploaded figures meet our requirements (or NAAS is able to fix the files to meet our requirements), the figure will be marked as "fixed" above. If NAAS is unable to fix the files, a red "failed" label will appear above. When NAAS has confirmed that the figure files meet our requirements, please download the file via the download option, and include these NAAS processed figure files when submitting your revised manuscript.

# Author comment: Author response to Decision Letter 1

**DOI:** 10.1371/journal.pmen.0000630.r002

21 Jan 2026

Attachment Submitted filename: response to PLOS MH reviews.docx

# Peer review: Decision Letter 1

**DOI:** 10.1371/journal.pmen.0000630.r003

**Authors:** Jason Morgan, Karli Montague-Cardoso

5 Apr 2026

PMEN-D-25-00516R1

Predicting and differentiating accidental and self-harm drug poisonings using health records data

PLOS Mental Health

Dear Dr. Simon,

Thank you for submitting your revised manuscript to PLOS Mental Health. As explained in a prior email, I have reached out to a second reviewer, who has now provided their comments, which you can access in the attached document. In light of these comments, I would like to request a final round of minor revisions. Thank you for your patience and understanding.

Please submit your revised manuscript by May 03 2026 11:59PM. If you will need more time than this to complete your revisions, please reply to this message or contact the journal office at mentalhealth@plos.org. When you're ready to submit your revision, log on to https://www.editorialmanager.com/pmen/ and select the 'Submissions Needing Revision' folder to locate your manuscript file.

Please include the following items when submitting your revised manuscript:

- A letter that responds to each point raised by the editor and reviewer(s). You should upload this letter as a separate file labeled 'Response to Reviewers'.
- A marked-up copy of your manuscript that highlights changes made to the original version. You should upload this as a separate file labeled 'Revised Manuscript with Track Changes'.
- An unmarked version of your revised paper without tracked changes. You should upload this as a separate file labeled 'Manuscript'.

Guidelines for resubmitting your figure files are available below the reviewer comments at the end of this letter.

As the corresponding author, your ORCID iD is verified in the submission system and will appear in the published article. PLOS supports the use of ORCID, and we encourage all coauthors to register for an ORCID iD and use it as well. Please encourage your coauthors to verify their ORCID iD within the submission system before final acceptance, as unverified ORCID iDs will not appear in the published article. *Only*  the individual author can complete the verification step; PLOS staff *cannot*  verify ORCID iDs on behalf of authors.

We look forward to receiving your revised manuscript.

Kind regards,

Karli Montague-Cardoso

Staff Editor

PLOS Mental Health

**Journal Requirements:**

If the reviewer comments include a recommendation to cite specific previously published works, please review and evaluate these publications to determine whether they are relevant and should be cited. There is no requirement to cite these works unless the editor has indicated otherwise.

Please review your reference list to ensure that it is complete and correct. If you have cited papers that have been retracted, please include the rationale for doing so in the manuscript text, or remove these references and replace them with relevant current references. Any changes to the reference list should be mentioned in the rebuttal letter that accompanies your revised manuscript. If you need to cite a retracted article, indicate the article’s retracted status in the References list and also include a citation and full reference for the retraction notice.

**Additional Editor Comments (if provided):**

[Note: HTML markup is below. Please do not edit.]

Reviewers' comments:

Reviewer's Responses to Questions

**Comments to the Author**

1. If the authors have adequately addressed your comments raised in a previous round of review and you feel that this manuscript is now acceptable for publication, you may indicate that here to bypass the “Comments to the Author” section, enter your conflict of interest statement in the “Confidential to Editor” section, and submit your "Accept" recommendation.

Reviewer #1: All comments have been addressed

Reviewer #2: (No Response)

**********

2. Does this manuscript meet PLOS Mental Health’s publication criteria? Is the manuscript technically sound, and do the data support the conclusions? The manuscript must describe methodologically and ethically rigorous research with conclusions that are appropriately drawn based on the data presented.

Reviewer #1: Yes

Reviewer #2: Yes

**********

3. Has the statistical analysis been performed appropriately and rigorously?

Reviewer #1: Yes

Reviewer #2: Yes

**********

4. Have the authors made all data underlying the findings in their manuscript fully available (please refer to the Data Availability Statement at the start of the manuscript PDF file)?

The PLOS Data policy requires authors to make all data underlying the findings described in their manuscript fully available without restriction, with rare exception. The data should be provided as part of the manuscript or its supporting information, or deposited to a public repository. For example, in addition to summary statistics, the data points behind means, medians and variance measures should be available. If there are restrictions on publicly sharing data—e.g. participant privacy or use of data from a third party—those must be specified.

Reviewer #1: Yes

Reviewer #2: Yes

**********

5. Is the manuscript presented in an intelligible fashion and written in standard English?

PLOS Mental Health does not copyedit accepted manuscripts, so the language in submitted articles must be clear, correct, and unambiguous. Any typographical or grammatical errors should be corrected at revision, so please note any specific errors here.

Reviewer #1: Yes

Reviewer #2: Yes

**********

6. Review Comments to the Author

Please use the space provided to explain your answers to the questions above. You may also include additional comments for the author, including concerns about dual publication, research ethics, or publication ethics. (Please upload your review as an attachment if it exceeds 20,000 characters)

**Reviewer #1:**  (No Response)

**Reviewer #2:**  Overall, this is a well-designed, highly rigorous retrospective cohort study. The use of a sequential, two-step modeling pipeline to parse apart spectral overlap in poisoning intent is a strong methodological contribution to the health informatics literature. Please see the attachment for my comments that may help the authors improve the manuscript.

**********

7. PLOS authors have the option to publish the peer review history of their article (what does this mean?). If published, this will include your full peer review and any attached files.

**Do you want your identity to be public for this peer review?**  If you choose “no”, your identity will remain anonymous but your review may still be made public.

For information about this choice, including consent withdrawal, please see our Privacy Policy.

Reviewer #1: **Yes:**  Jacques Eugene Raubenheimer

Reviewer #2: No

**********

[NOTE: If reviewer comments were submitted as an attachment file, they will be attached to this email and accessible via the submission site. Please log into your account, locate the manuscript record, and check for the action link "View Attachments". If this link does not appear, there are no attachment files.]

**Figure Resubmissions:**

While revising your submission, we strongly recommend that you use PLOS’s NAAS tool (https://ngplosjournals.pagemajik.ai/artanalysis) to test your figure files. NAAS can convert your figure files to the TIFF file type and meet basic requirements (such as print size, resolution), or provide you with a report on issues that do not meet our requirements and that NAAS cannot fix.

After uploading your figures to PLOS’s NAAS tool - https://ngplosjournals.pagemajik.ai/artanalysis, NAAS will process the files provided and display the results in the "Uploaded Files" section of the page as the processing is complete. If the uploaded figures meet our requirements (or NAAS is able to fix the files to meet our requirements), the figure will be marked as "fixed" above. If NAAS is unable to fix the files, a red "failed" label will appear above. When NAAS has confirmed that the figure files meet our requirements, please download the file via the download option, and include these NAAS processed figure files when submitting your revised manuscript.

# Author comment: Author response to Decision Letter 2

**DOI:** 10.1371/journal.pmen.0000630.r004

7 May 2026

Attachment Submitted filename: response to 2nd PLOS review.docx

# Editor report: Decision Letter 2

**DOI:** 10.1371/journal.pmen.0000630.r005

**Authors:** Jason Morgan, Karli Montague-Cardoso, Karli Montague-Cardoso

12 May 2026

Predicting and differentiating accidental and self-harm drug poisonings using health records data

PMEN-D-25-00516R2

Dear Dr. Simon,

We are pleased to inform you that your manuscript 'Predicting and differentiating accidental and self-harm drug poisonings using health records data' has been provisionally accepted for publication in PLOS Mental Health.

Before your manuscript can be formally accepted you will need to complete some formatting changes, which you will receive in a follow up email. A member of our team will be in touch with a set of requests.

Please note that your manuscript will not be scheduled for publication until you have made the required changes, so a swift response is appreciated.

IMPORTANT: The editorial review process is now complete. PLOS will only permit corrections to spelling, formatting or significant scientific errors from this point onwards. Requests for major changes, or any which affect the scientific understanding of your work, will cause delays to the publication date of your manuscript.

If your institution or institutions have a press office, please notify them about your upcoming paper to help maximize its impact. If they'll be preparing press materials, please inform our press team as soon as possible -- no later than 48 hours after receiving the formal acceptance. Your manuscript will remain under strict press embargo until 2 pm Eastern Time on the date of publication. For more information, please contact mentalhealth@plos.org.

Thank you again for supporting Open Access publishing; we are looking forward to publishing your work in PLOS Mental Health.

Best regards,

Karli Montague-Cardoso

Staff Editor

PLOS Mental Health

***********************************************************

Reviewer Comments (if any, and for reference):
